# Association of obesity with heart failure outcomes in 11 Asian regions: A cohort study

**DOI:** 10.1371/journal.pmed.1002916

**Published:** 2019-09-24

**Authors:** Chanchal Chandramouli, Wan Ting Tay, Nurul Sahiddah Bamadhaj, Jasper Tromp, Tiew-Hwa Katherine Teng, Jonathan J. L. Yap, Michael R. MacDonald, Chung-Lieh Hung, Koen Streng, Ajay Naik, Gurpreet Singh Wander, Jitendra Sawhney, Lieng Hsi Ling, A. Mark Richards, Inder Anand, Adriaan A. Voors, Carolyn S. P. Lam, on behalf of ASIAN-HF Investigators

**Affiliations:** 1 National Heart Centre Singapore, Singapore, Singapore; 2 University Medical Center Groningen, Groningen, Netherlands; 3 Changi General Hospital, Singapore, Singapore; 4 MacKay Memorial Hospital, Taipei, Taiwan; 5 CIMS Hospital, Ahmedabad, Gujarat, India; 6 Dayanand Medical College and Hospital, Ludhiana, Punjab, India; 7 Sir Gangaram Hospital, New Delhi, India; 8 Cardiovascular Research Institute, Singapore, Singapore; 9 Veterans Affairs Medical Center, Minneapolis, Minnesota, United States of America; 10 Duke-NUS Medical School, Singapore, Singapore; University of Oxford, UNITED KINGDOM

## Abstract

**Background:**

Asians are predisposed to a lean heart failure (HF) phenotype. Data on the ‘obesity paradox’, reported in Western populations, are scarce in Asia and have only utilised the traditional classification of body mass index (BMI). We aimed to investigate the association between obesity (defined by BMI and abdominal measures) and HF outcomes in Asia.

**Methods and findings:**

Utilising the Asian Sudden Cardiac Death in Heart Failure (ASIAN-HF) registry (11 Asian regions including Taiwan, Hong Kong, China, India, Malaysia, Thailand, Singapore, Indonesia, Philippines, Japan, and Korea; 46 centres with enrolment between 1 October 2012 and 6 October 2016), we prospectively examined 5,964 patients with symptomatic HF (mean age 61.3 ± 13.3 years, 26% women, mean BMI 25.3 ± 5.3 kg/m^2^, 16% with HF with preserved ejection fraction [HFpEF; ejection fraction ≥ 50%]), among whom 2,051 also had waist-to-height ratio (WHtR) measurements (mean age 60.8 ± 12.9 years, 24% women, mean BMI 25.0 ± 5.2 kg/m^2^, 7% HFpEF). Patients were categorised by BMI quartiles or WHtR quartiles or 4 combined groups of BMI (low, <24.5 kg/m^2^ [lean], or high, ≥24.5 kg/m^2^ [obese]) and WHtR (low, <0.55 [thin], or high, ≥0.55 [fat]). Cox proportional hazards models were used to examine a 1-year composite outcome (HF hospitalisation or mortality). Across BMI quartiles, higher BMI was associated with lower risk of the composite outcome (*p*_trend_ < 0.001). Contrastingly, higher WHtR was associated with higher risk of the composite outcome. Individuals in the lean-fat group, with low BMI and high WHtR (13.9%), were more likely to be women (35.4%) and to be from low-income countries (47.7%) (predominantly in South/Southeast Asia), and had higher prevalence of diabetes (46%), worse quality of life scores (63.3 ± 24.2), and a higher rate of the composite outcome (51/232; 22%), compared to the other groups (*p <* 0.05 for all). Following multivariable adjustment, the lean-fat group had higher adjusted risk of the composite outcome (hazard ratio 1.93, 95% CI 1.17–3.18, *p =* 0.01), compared to the obese-thin group, with high BMI and low WHtR. Results were consistent across both HF subtypes (HFpEF and HF with reduced ejection fraction [HFrEF]; *p*_interaction_ = 0.355). Selection bias and residual confounding are potential limitations of such multinational observational registries.

**Conclusions:**

In this cohort of Asian patients with HF, the ‘obesity paradox’ is observed only when defined using BMI, with WHtR showing the opposite association with the composite outcome. Lean-fat patients, with high WHtR and low BMI, have the worst outcomes. A direct correlation between high WHtR and the composite outcome is apparent in both HFpEF and HFrEF.

**Trial registration:**

Asian Sudden Cardiac Death in HF (ASIAN-HF) Registry ClinicalTrials.gov Identifier: NCT01633398

## Abstract 2

Carolyn Lam and colleagues investigate the association of body mass index and waist-to-height ratios with heart failure outcomes in cohorts from 11 Asian regions.

## Author summary

**Why was this study done?:**

- Asian heart failure (HF) patients are more likely to have a lean HF phenotype compared to Western patients.
- The obesity paradox, where higher body mass index (BMI) is associated with better HF outcomes, is well-reported in Western populations, but little is known about this paradox in Asia.
- We investigated the association between obesity, defined by traditional BMI versus waist circumference indices, and HF outcomes in Asia.

**What did the researchers do and find?:**

- Among Asian patients with HF, BMI and waist-to-height ratio (WHtR) have directionally opposite relationships with a composite outcome of all-cause death and hospitalisation for HF—lower BMI, but higher WHtR, is associated with worse outcomes.
- In combined analysis, patients who were lean-fat (with low BMI but high WHtR) were more likely to be women, to be from low-income countries or South/Southeast Asia, to have diabetes, and to have worse quality of life scores.
- Lean-fat patients also had the highest rate and risk of the composite outcome (HF hospitalisation and all-cause mortality), irrespective of heart failure subtype (with preserved versus reduced ejection fraction).

**What do these findings mean?:**

- The obesity paradox exists in Asia only when obesity is classified by BMI.
- The relationship between obesity and HF outcomes in Asia is directionally opposite for BMI versus WHtR.
- In this cohort of Asian patients with HF, lean (low BMI) patients with central obesity (high WHtR) had the worst outcomes, both in cases where ejection fraction was reduced and in cases where it was preserved.

## Introduction

Obesity poses a major public health challenge globally, affecting 650 million worldwide, with a worrisome tripling of its prevalence over the last 20 years, especially among young adults in low- and middle-income countries [1]. Ethnic differences in the association of body mass index (BMI) with clinical outcomes have been observed in the general population, with Asians being at higher risk of adverse events at lower BMI cutoffs compared to international standards [2]. WHO has therefore recommended lower BMI cutoffs for the definition of obesity and clinical preventive efforts among Asians [3].

Obesity is an antecedent risk factor of heart failure (HF). In established HF, however, a paradoxical relationship between obesity and outcomes has been described, where obese HF patients (defined by higher BMI) have better outcomes compared to those with lower BMI. This ‘obesity paradox’ was described mainly in Western cohorts of patients with HF with preserved ejection fraction (HFpEF) or HF with reduced ejection fraction (HFrEF) [4–6], and in limited Asian populations with HFrEF [7,8]. Of note, we recently showed that Asians, particularly in Southeast Asia, are predisposed to a unique lean diabetic HFpEF phenotype [9], with the worst outcomes among all HF phenotypes, including ischemic and non-ischemic HFrEF. Moreover, BMI is known to be a remarkably heterogeneous parameter and does not account for body fat distribution [10]. Consideration of body fat distribution has gained momentum in recent years, with obesity measures other than BMI—including waist circumference, waist-to-hip ratio, waist-to-height ratio (WHtR), and body fat percentage—having better discriminatory capacity than BMI in predicting cardiovascular outcomes [11,12]. Findings from TOPCAT (Treatment of Preserved Cardiac Function Heart Failure with an Aldosterone Antagonist) showed greater risk of mortality among HFpEF patients with higher waist circumference (≥102 cm in men, ≥88 cm in women) [13]. Similarly, in BIOSTAT-CHF (BIOlogy Study to TAilored Treatment in Chronic Heart Failure), higher waist-to-hip ratio was linked to higher risk of death in women [14]. There is a lack of data on waist circumference measurements in Asian patients with HF, and prior data using BMI are limited to HFrEF in Asia. Therefore, we sought to examine the association of obesity, defined by BMI versus waist circumference, with HF outcomes in patients with HFpEF and HFrEF in Asia.

## Methods

### Study design, study population, and setting

This study adheres to the Strengthening the Reporting of Observational Studies in Epidemiology (STROBE) guidelines (S1 Checklist). Ethics approvals were obtained from the relevant human ethics committees at all sites (S1 Table). The study complied with the Declaration of Helsinki, and all patients provided written informed consent.

We studied the association of obesity with outcomes using the Asian Sudden Cardiac Death in Heart Failure (ASIAN-HF) registry [2,15]. The prospective study design, with details on sample size, of the ASIAN-HF registry has been published previously [15]. Briefly, ASIAN-HF is a prospective observational multinational registry with patients with HF from 46 investigation sites in 11 Asian regions (Taiwan, Hong Kong, China, India, Malaysia, Thailand, Singapore, Indonesia, Philippines, Japan, and Korea) that enrolled patients between 1 October 2012 and 6 October 2016 (S1 Text). The broad purpose of the ASIAN-HF registry was to determine the characteristics, mortality/morbidity, and risk factors for adverse outcomes among Asian patients with symptomatic HF (stage C). Although the specific primary analysis described in the study design [15] related to investigating sudden cardiac death in HFrEF [16], prespecified aims also included examining risk factors for outcomes (including both deaths and hospitalisations), as well as assessing the thresholds of traditional risk factors that are associated with increased risk in Asian patients.

The recruitment of patients with HFpEF was delayed by 1 year (9 September 2013 versus 1 October 2012) relative to the recruitment of patients with HFrEF, for funding reasons. For most of the recruitment period (until 6 October 2016), there was overlap in recruitment of both types of HF, and no apparent shifts in epidemiology were expected. Feasibility of follow-up throughout the duration of the study through periodic contact was ensured. The key inclusion criteria were >18 years of age with symptomatic HF (at least 1 previous episode of decompensated HF in the previous 6 months resulting in a hospital admission or treatment in an outpatient clinic). Patients with severe valvular heart disease as a cause of HF, with a life-threatening comorbidity with a life expectancy < 1 year, or who were unable or unwilling to give consent were excluded. All patients underwent physical examination at baseline: Waist circumference was measured in *n* = 2,051 patients, and BMI measurements were available for the entire cohort (*n* = 5,964).

### Study definitions

**Fig 1:**
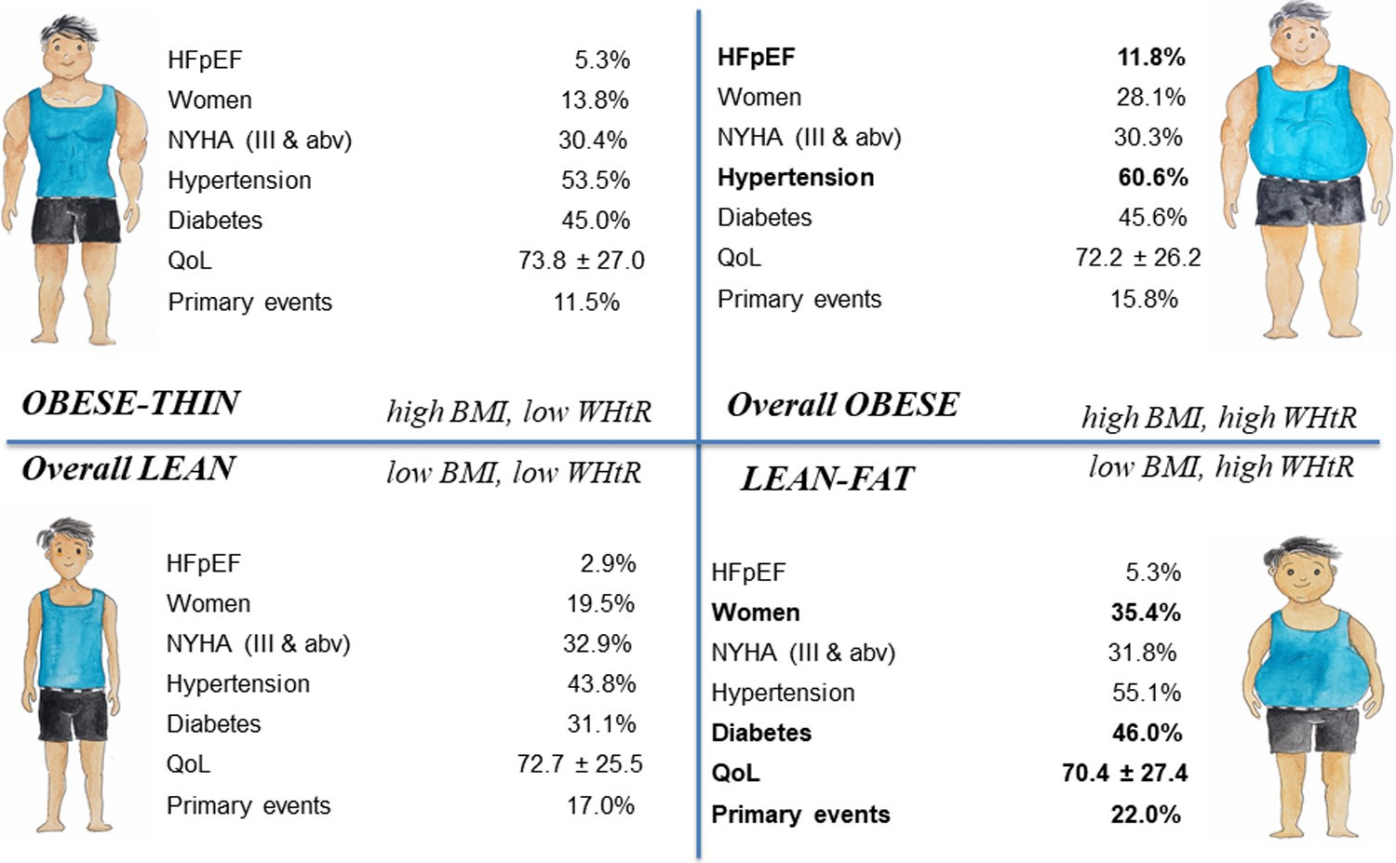
Central illustration of 4 combined groups of BMI (high, ≥24.5 kg/m^2^ [obese], or low, <24.5 kg/m^2^ [lean]) and waist-to-height ratio (high, ≥0.55 [fat], or low, <0.55 [thin]). QoL refers to the Kansas City Cardiomyopathy Questionnaire total symptom score. Key differences in characteristics between BMI/WHtR groups in bold. abv, above; BMI, body mass index; HFpEF, heart failure with preserved ejection fraction; NYHA, New York Heart Association; QoL, quality of life; WHtR, waist-to-height ratio.

Patients were grouped by (1) BMI quartiles (<21.8, 21.8 to <24.5, 24.5 to <27.8, and ≥27.8 kg/m^2^), (2) WHtR quartiles (<0.51, 0.51 to <0.55, 0.55 to <0.60, and ≥0.60), and (3) 4 combined anthropometric groups of BMI (high, ≥24.5 kg/m^2^ [obese], or low, <24.5 kg/m^2^ [lean]) and WHtR (high, ≥0.55 [fat], or low, <0.55 [thin]) (Fig 1).

The definitions of comorbidities in ASIAN-HF have been previously described [2,15]. Coronary artery disease was defined as significant coronary obstruction on angiography, history of myocardial infarction, or prior revascularization. Hypertension was defined as the clinical diagnosis (blood pressure ≥ 140/90 mm Hg) and/or receiving anti-hypertensive medication. Diabetes was defined as prior history of diabetes and/or receiving anti-diabetic therapy. The Modification of Diet in Renal Disease (MDRD) Study equation was utilised to compute estimated glomerular filtration rate (eGFR), and chronic kidney disease was defined as eGFR < 60 ml/min/1.73 m^2^. Health status was measured using the Kansas City Cardiomyopathy Questionnaire (KCCQ), a validated 23-item patient-centred HF-specific questionnaire [17]. Higher KCCQ domain scores (0 to 100) represent better health status.

### Body composition analysis

In a group of HF patients recruited in Singapore (*n* = 311), bioelectrical impedance analysis was performed (InBody370; InBody, Cerritos, CA, US). Body weight, skeletal muscle mass, fat mass, and estimated trunk fat were measured using the direct segmental (right arm, left arm, trunk, right leg, left leg) method at 3 different frequencies (5 kHz, 50 kHz, and 250 kHz), as described previously [18]. The variables trunk fat, skeletal muscle, and fat mass were calculated as percentage of body weight. In separate regression models, each of these body composition parameters was treated as the dependent variable, while waist-to-hip ratio was the independent variable, with the model adjusted for age, sex, and BMI.

### Outcomes

Patients were followed up for protocol-defined outcomes, which were adjudicated by an independent clinical endpoint committee. The primary outcome was the composite of 1-year all-cause mortality or hospitalisation for HF. Secondary outcomes were 1-year all-cause mortality, 1-year HF hospitalisation, 1-year cardiovascular mortality, and health-related quality of life (QoL) assessed with the KCCQ assessed at baseline. As a sensitivity analysis, the association of BMI and outcomes was also examined using WHO-recommended Asian cutoffs (<18.5, 18.5 to <23.0, 23.0 to <27.5, and ≥27.5 kg/m^2^ for underweight, normal, overweight, and obese, respectively) in the whole cohort with follow-up data (*n* = 5,397). All data were prospectively documented in an electronic database capture, which was managed by a contract research organisation (IQVIA, formerly Quintiles) appointed by the academic executive committee.

### Statistical analysis

Baseline characteristics for the groups of patients were described using frequencies with percentages for categorical variables and means with standard deviations for continuous variables. Differences between groups were tested using Pearson χ² tests for categorical variables and ANOVA for continuous variables. A test of trend across the quartiles of WHtR and BMI was also performed. Restricted cubic regression splines were used to examine the association of BMI and WHtR with the composite outcome. Time-to-event analyses were examined using multivariable Cox proportional hazards models in the absence of violation of the proportional hazards assumption. Adjustments included age, sex, ethnicity, enrolment type (recruited during inpatient stay versus at outpatient clinic), New York Heart Association (NYHA) class, systolic blood pressure, heart rate, ejection fraction (EF), history of coronary artery disease, atrial fibrillation, diabetes, peripheral oedema, peripheral arterial vascular disease, and use of an angiotensin-converting enzyme inhibitor (ACEi)/angiotensin receptor blocker (ARB), beta blocker, diuretic, or mineralocorticoid receptor antagonist (MRA). The primary endpoint of 1-year all-cause mortality or HF hospitalisation was censored at 1 year or the last date of follow-up, whichever was earlier, in patients who did not have an event. To determine if HF subtype (i.e., HFrEF [EF < 40%] or HFpEF [EF ≥ 50%]) modified the relationship between obesity and HF outcomes, the interaction between HF subtype and BMI/WHtR/combined anthropometric groups, adjusted for age, was examined in the Cox model. Sensitivity analysis of the relationship between BMI and HF outcomes was performed in the entire cohort. Marginal KCCQ domain scores, adjusted for age, sex, ethnicity, enrolment type, education, NYHA class, systolic blood pressure, heart rate, HF subtype, history of coronary artery disease, atrial fibrillation, and diabetes, were examined. In view of a reviewer’s suggestion, sex-stratified analysis of the association of WHtR with HF outcomes was also performed.

Patients with incomplete data on adjusted clinical and demographic factors were excluded from multivariable logistic and Cox regression analyses. All analyses were performed with Stata/SE version 14.0 (StataCorp, College Station, TX, US).

## Results

### Baseline characteristics

In the entire cohort of 5,964 Asian patients with HF (mean age 61.3 ± 13.3 years, 26% women, 16% HFpEF), with increasing BMI category (WHO-recommended Asian cutoffs), patients were younger, with higher blood pressure, larger waist measures, greater prevalence of comorbidities (hypertension, diabetes and peripheral oedema), and higher QoL scores (*p*_trend_ < 0.05; S2 Table).

Asian patients with HF with (versus without) waist measures available were marginally younger (60.8 ± 12.9 versus 61.6 ± 13.5 years, *p =* 0.018), had a lower prevalence of HFpEF (6.8% versus 20.8%), belonged to less severe NYHA classes (68.5% versus 66.2%), had similar heart rate (78.8 ± 14.8 versus 78.8 ± 16.2 bpm), had similar BMI (25.0 ± 5.2 versus 25.4 ± 5.4 kg/m^2^), and had similar prevalence of both chronic kidney disease (43.7% versus 46.4%) and diabetes (40.4% versus 41.7%) (S3 Table; *p >* 0.05 for all).

**Fig 2:**
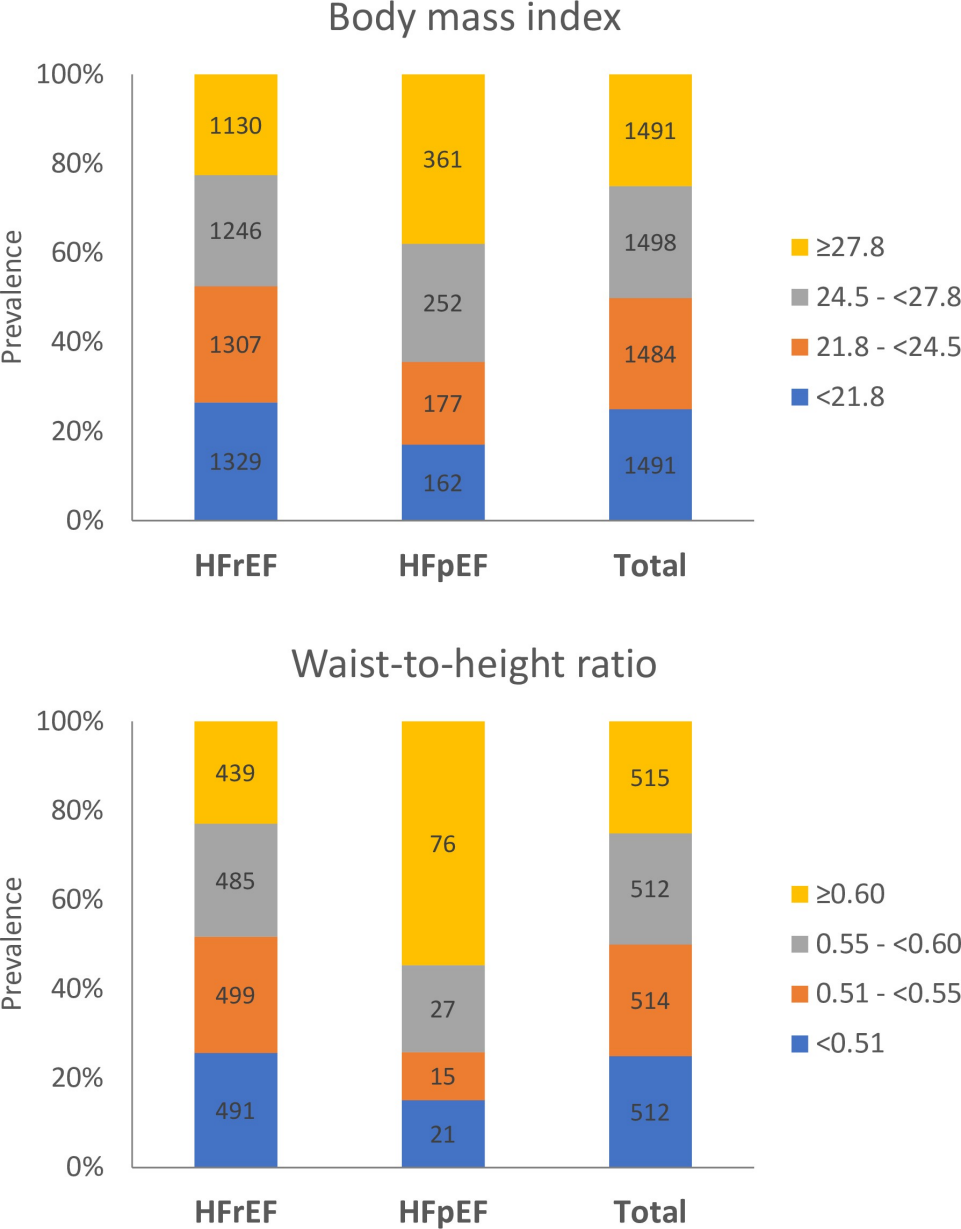
Prevalence of HFpEF and HFrEF stratified by body mass index and waist-to-height ratio. HFpEF, heart failure with preserved ejection fraction; HFrEF, heart failure with reduced ejection fraction.

Among 2,051 Asian patients with HF who had both BMI and WHtR measurements (mean age 60.8 ± 12.9 years, 24% women, 7% HFpEF), mean BMI and waist circumference were 25.0 ± 5.2 kg/m^2^ and 91.1 ± 12.5 cm; the 5th and 95th percentiles of BMI and WHtR were 18.3–34.2 kg/m^2^ and 0.45–0.70, respectively. Patients with HFpEF, compared to HFrEF, had higher mean BMI (27.1 ± 6.0 versus 24.2 ± 5.1 kg/m^2^) and waist circumference (98.2 ± 16.5 versus 90.7 ± 12.0 cm) (*p <* 0.001 for both). Notably, half of patients with HFpEF (54.7%) had abdominal obesity (WHtR > 0.60), in contrast to a quarter of HFrEF patients (23%) (S4 Table and Fig 2).

Results with increasing BMI quartiles when restricted to patients with waist measures (*n =* 2,051; S4 Table) were similar to those in the larger cohort (*n =* 5,964; S2 Table), as described above. Larger WHtR (increasing WHtR quartiles) was associated with a higher proportion of women, higher BMI, and higher prevalence of hypertension, diabetes, and peripheral oedema (*p*_trend_ < 0.001; S4 Table). The highest WHtR quartile was associated with the lowest QoL overall summary score (63.3 ± 23.5), more so in HFpEF than HFrEF (*p*_interaction_ = 0.037).

In combined analyses of BMI and WHtR (Table 1), lean-fat (low BMI/high WHtR) patients (13.9%) had the highest proportion of women (35.4%), hypertension (55.1%), and diabetes (46.0%); were more likely to be from low-income countries or to be from South Asia or Southeast Asia; and had the lowest QoL scores (5–9 points lower than other groups), with respect to the self-efficacy (63.7 ± 29.1) and social limitation (59.9 ± 32.7) domains (*p <* 0.05).

**Table 1:** Baseline characteristics and 1-year outcomes by BMI and WHtR groups.

Characteristic	Obese-thin (high BMI, low WHtR)	Overall obese (high BMI, high WHtR)	Overall lean (low BMI, low WHtR)	Lean-fat (low BMI, high WHtR)	*p*-Value (ANOVA/χ^2^)
***n***	283	745	738	285	
**HFpEF**	15 (5.3%)	88 (11.8%)	21 (2.9%)	15 (5.3%)	**<0.001**
**Demographics**					
Age, years	58.9 (12.7)	59.5 (12.6)	61.5 (13.4)	63.9 (11.9)	<0.001
Women	39 (13.8%)	209 (28.1%)	144 (19.5%)	101 (35.4%)	<0.001
Ethnicity					0.011
Chinese	57 (20.1%)	177 (23.8%)	175 (23.7%)	66 (23.2%)	
Indian	113 (39.9%)	308 (41.3%)	279 (37.8%)	123 (43.2%)	
Malay	42 (14.8%)	86 (11.5%)	77 (10.4%)	38 (13.3%)	
Japanese/Korean	23 (8.1%)	56 (7.5%)	93 (12.6%)	32 (11.2%)	
Other (Thai, Filipino, indigenous)	48 (17.0%)	118 (15.8%)	114 (15.4%)	26 (9.1%)	
Geographical region, percent					0.028
Northeast Asia	56 (19.8%)	130 (17.4%)	180 (24.4%)	51 (17.9%)	
South Asia	98 (34.6%)	279 (37.4%)	261 (35.4%)	114 (40.0%)	
Southeast Asia	129 (45.6%)	336 (45.1%)	297 (40.2%)	120 (42.1%)	
Country-level economic development					<0.001
Low income	111 (39.2%)	316 (42.4%)	304 (41.2%)	136 (47.7%)	
Middle income	118 (41.7%)	217 (29.1%)	236 (32.0%)	52 (18.2%)	
High income	54 (19.1%)	212 (28.5%)	198 (26.8%)	97 (34.0%)	
**Clinical characteristics**					
NYHA					0.740
Class I or II	179 (69.6%)	483 (69.7%)	467 (67.1%)	187 (68.2%)	
Class III or IV	78 (30.4%)	210 (30.3%)	229 (32.9%)	87 (31.8%)	
Systolic blood pressure, mm Hg	120.2 (18.6)	122.7 (20.2)	115.0 (18.9)	120.3 (19.3)	<0.001
Diastolic blood pressure, mm Hg	73.5 (11.7)	74.1 (12.2)	71.2 (11.3)	71.4 (11.5)	<0.001
Heart rate, bpm	78.9 (15.9)	79.3 (14.8)	78.8 (15.0)	77.7 (13.0)	0.500
BMI, kg/m^2^	26.6 (2.6)	29.6 (5.1)	21.0 (2.1)	22.1 (1.8)	<0.001
eGFR, ml/min/1.73 m^2^	64.4 (26.1)	65.8 (28.4)	65.9 (26.8)	64.1 (29.5)	0.770
**Comorbidities**					
Chronic kidney disease (eGFR < 60 ml/min/1.73 m^2^)	85 (45.0%)	252 (44.4%)	231 (40.7%)	113 (47.7%)	0.280
Ischemic HF	139 (49.1%)	351 (47.1%)	366 (49.7%)	144 (50.5%)	0.610
Hypertension	151 (53.5%)	451 (60.6%)	322 (43.8%)	157 (55.1%)	<0.001
Coronary artery disease	145 (51.4%)	380 (51.1%)	384 (52.2%)	152 (53.3%)	0.920
Atrial fibrillation	31 (11.0%)	119 (16.0%)	118 (16.0%)	48 (16.8%)	0.170
Myocardial infarction	91 (32.3%)	235 (31.6%)	234 (31.8%)	90 (31.6%)	0.997
Diabetes	127 (45.0%)	339 (45.6%)	229 (31.1%)	131 (46.0%)	<0.001
Prior stroke	16 (5.7%)	55 (7.4%)	36 (4.9%)	25 (8.8%)	0.076
Peripheral artery vascular disease	6 (2.1%)	19 (2.6%)	22 (3.0%)	10 (3.5%)	0.740
Peripheral oedema	55 (19.4%)	187 (25.1%)	125 (17.0%)	55 (19.3%)	0.001
COPD	16 (5.7%)	53 (7.1%)	54 (7.3%)	23 (8.1%)	0.720
Smoking, ever	134 (47.5%)	318 (42.7%)	347 (47.2%)	109 (38.2%)	0.034
Alcohol, ever	78 (27.7%)	215 (28.9%)	215 (29.3%)	67 (23.5%)	0.290
**Medications**					
ACEi or ARB	209 (74.9%)	543 (74.4%)	513 (70.8%)	188 (68.9%)	0.170
Beta blocker	211 (75.6%)	569 (77.9%)	530 (73.1%)	194 (71.1%)	0.070
Diuretic	207 (74.2%)	632 (86.6%)	599 (82.6%)	225 (82.4%)	<0.001
MRA	149 (53.4%)	379 (51.9%)	406 (56.0%)	134 (49.1%)	0.200
**Outcomes at 1 year**					
All-cause mortality	19 (8.1%)	62 (9.4%)	73 (11.7%)	36 (15.5%)	0.031
Cardiovascular mortality	18 (7.8%)	52 (8.1%)	63 (10.6%)	33 (14.5%)	0.026
Composite outcome (all death + HF hospitalisation)	27 (11.5%)	103 (15.6%)	104 (16.7%)	51 (22.0%)	0.022
**Quality of life at baseline (*n =* 1,990)**					
Physical limitation score	70.3 (25.2)	71.5 (24.4)	71.2 (25.6)	67.8 (27.5)	0.210
Symptom stability score	62.4 (26.7)	59.8 (27.3)	61.4 (27.7)	64.3 (28.1)	0.110
Symptom frequency score	72.3 (28.3)	70.0 (28.3)	71.5 (27.0)	69.1 (28.6)	0.430
Symptom burden score	75.4 (27.4)	74.5 (26.2)	74.0 (25.6)	71.7 (27.7)	0.380
Total symptom score	73.8 (27.0)	72.2 (26.2)	72.7 (25.5)	70.4 (27.4)	0.470
Self-efficacy score	68.9 (26.4)	67.7 (27.3)	64.7 (27.4)	63.7 (29.1)	0.027
Social limitation score	68.8 (30.0)	65.4 (31.5)	63.2 (33.2)	59.9 (32.7)	0.011
Overall summary score	67.7 (22.4)	66.4 (22.6)	65.6 (23.0)	63.3 (24.2)	0.130
Clinical summary score	72.1 (23.3)	71.9 (22.5)	72.0 (22.8)	69.1 (24.9)	0.300

Data given as mean (standard deviation) or number (percent).

ACEi, angiotensin-converting enzyme inhibitor; ARB, angiotensin receptor blocker; BMI, body mass index; COPD, chronic obstructive pulmonary disease; eGFR, estimated glomerular filtration rate; HF, heart failure; HFpEF, heart failure with preserved ejection fraction; MRA, mineralocorticoid receptor antagonist; NYHA, New York Heart Association; WHtR, waist-to-height ratio.

Overall obese (high BMI/high WHtR) patients (36.3%) were more likely to have HFpEF (11.8%), to be from South Asia/Southeast Asia, and to have hypertension (60.6%) and peripheral oedema (25.1%) (*p <* 0.001 for all; Table 1).

Overall lean (low BMI/low WHtR) patients (36.0%) were more likely to have HFrEF (97.1%) and to be from Northeast Asia (24.4%), and had the lowest prevalence of comorbidities (hypertension, 43.8%; diabetes, 31.1%), compared to the other groups (*p <* 0.001).

Obese-thin (high BMI/low WHtR) patients (13.8%) were younger, were more often men, and had the highest QoL scores, compared to the other groups. The obese-thin patients also had lower prevalence of hypertension (53.5% versus 60.6%), compared to the overall obese (*p <* 0.05; Table 1).

### Body composition analysis

Among the 311 patients with additional bioelectrical impedance data, baseline characteristics were similar to the overall cohort (S5 Table). Patients who were overall obese (high BMI/high waist-to-hip ratio) had the highest percentage fat mass and trunk fat, followed by obese-thin (high BMI/low waist-to-hip ratio), lean-fat (low BMI/high waist-to-hip ratio), and overall lean (low BMI/low waist-to-hip ratio) (*p <* 0.001; S5 Table). Controlled for BMI, sex, and age, a 1-SD increase in waist-to-hip ratio was associated with a 0.38-unit increase in percent trunk fat (*p <* 0.05).

### Clinical outcomes and quality of life

Of the 2,051 Asian patients with both BMI and WHtR measurements available, follow-up data were complete in 1,746 (85.1%) patients, of whom 285 (16.3%) experienced the primary composite outcome of all-cause mortality or hospitalisation for HF within 1 year, and 190 (10.9%) patients died within 1 year.

**Fig 3:**
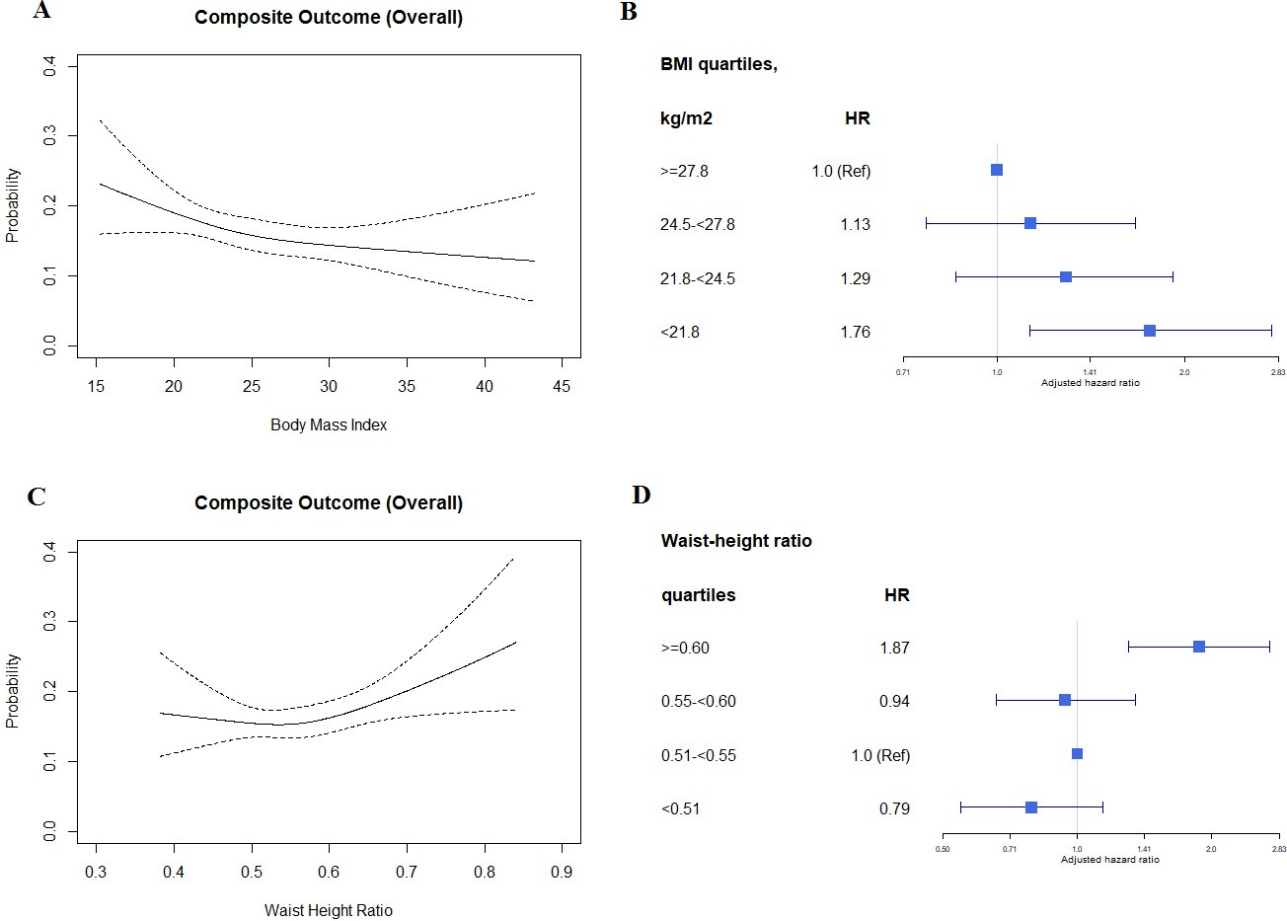
Association of BMI and waist-to-height ratio with the composite outcome using cubic spline regression models and Cox proportional hazards models. Cubic spline regression models (A and C); Cox proportional hazards models (B and D). HR, hazard ratio.

Cubic spline regression models showed an inverse relationship between BMI and risk of the composite outcome; in contrast, there was a direct relationship between WHtR and risk of the composite outcome. The associations of BMI and WHtR with the composite outcome were not modified by HF subtype (i.e., HFpEF or HFrEF; *p*_interaction_ = 0.760 and 0.881 respectively; Table 2). Our sensitivity analysis showed similar results for BMI when the entire cohort with follow-up data was included (*n =* 5,397; S6 Table) and when using WHO-recommended Asian BMI cutoffs (Fig 3).

**Table 2:** Association of BMI and WHtR with outcomes.

Outcome by BMI and WHtR	Number at risk	Number of events, *n* (%)	Unadjusted hazard ratio (95% CI)	*p*-Value	Adjusted* hazard ratio (95% CI)	*p*-Value
**1-year composite outcome**						
BMI and WHtR				0.017^†^		0.058^†^
Obese-thin (high BMI, low WHtR)	234	27 (11.5%)	1.00 (Reference)		1.00 (Reference)	
Overall obese (high BMI, high WHtR)	658	103 (15.6%)	1.40 (0.92, 2.14)	0.121	1.52 (0.97, 2.40)	0.068
Overall lean (low BMI, low WHtR)	622	104 (16.7%)	1.52 (1.00, 2.32)	0.051	1.63 (1.03, 2.56)	0.035
Lean-fat (low BMI, high WHtR)	232	51 (22.0%)	2.07 (1.30, 3.29)	0.002	1.93 (1.17, 3.18)	0.010
**1-year all-cause mortality**						
BMI and WHtR				0.025^†^		0.034^†^
Obese-thin (high BMI, low WHtR)	234	19 (8.1%)	1.00 (Reference)		1.00 (Reference)	
Overall obese (high BMI, high WHtR)	658	62 (9.4%)	1.17 (0.70, 1.96)	0.545	1.21 (0.70, 2.09)	0.504
Overall lean (low BMI, low WHtR)	622	73 (11.7%)	1.49 (0.90, 2.47)	0.121	1.67 (0.97, 2.87)	0.065
Lean-fat (low BMI, high WHtR)	232	36 (15.5%)	2.04 (1.17, 3.55)	0.012	2.01 (1.11, 3.65)	0.022
**1-year HF hospitalisation**						
BMI and WHtR				0.103^†^		0.207^†^
Obese-thin (high BMI, low WHtR)	234	10 (4.3%)	1.00 (Reference)		1.00 (Reference)	
Overall obese (high BMI, high WHtR)	658	50 (7.6%)	1.81 (0.92,3.57)	0.088	1.84 (0.90, 3.79)	0.096
Overall lean (low BMI, low WHtR)	622	45 (7.2%)	1.72 (0.87, 3.42)	0.120	1.59 (0.78, 3.42)	0.203
Lean-fat (low BMI, high WHtR)	232	24 (10.3%)	2.51 (1.20, 5.24)	0.015	1.99 (0.91, 4.35)	0.083

*Adjusted for age, sex, ethnicity, enrolment type, New York Heart Association class, systolic blood pressure, heart rate, ejection fraction, history of coronary artery disease, atrial fibrillation, diabetes, peripheral oedema, peripheral artery vascular disease, and use of angiotensin-converting enzyme inhibitor/angiotensin receptor blocker, beta blocker, diuretic, and mineralocorticoid receptor antagonist.

^†^Overall type III *p*-values for BMI/WHtR groups.

BMI, body mass index; WHtR, waist-to-height ratio.

**Fig 4:**
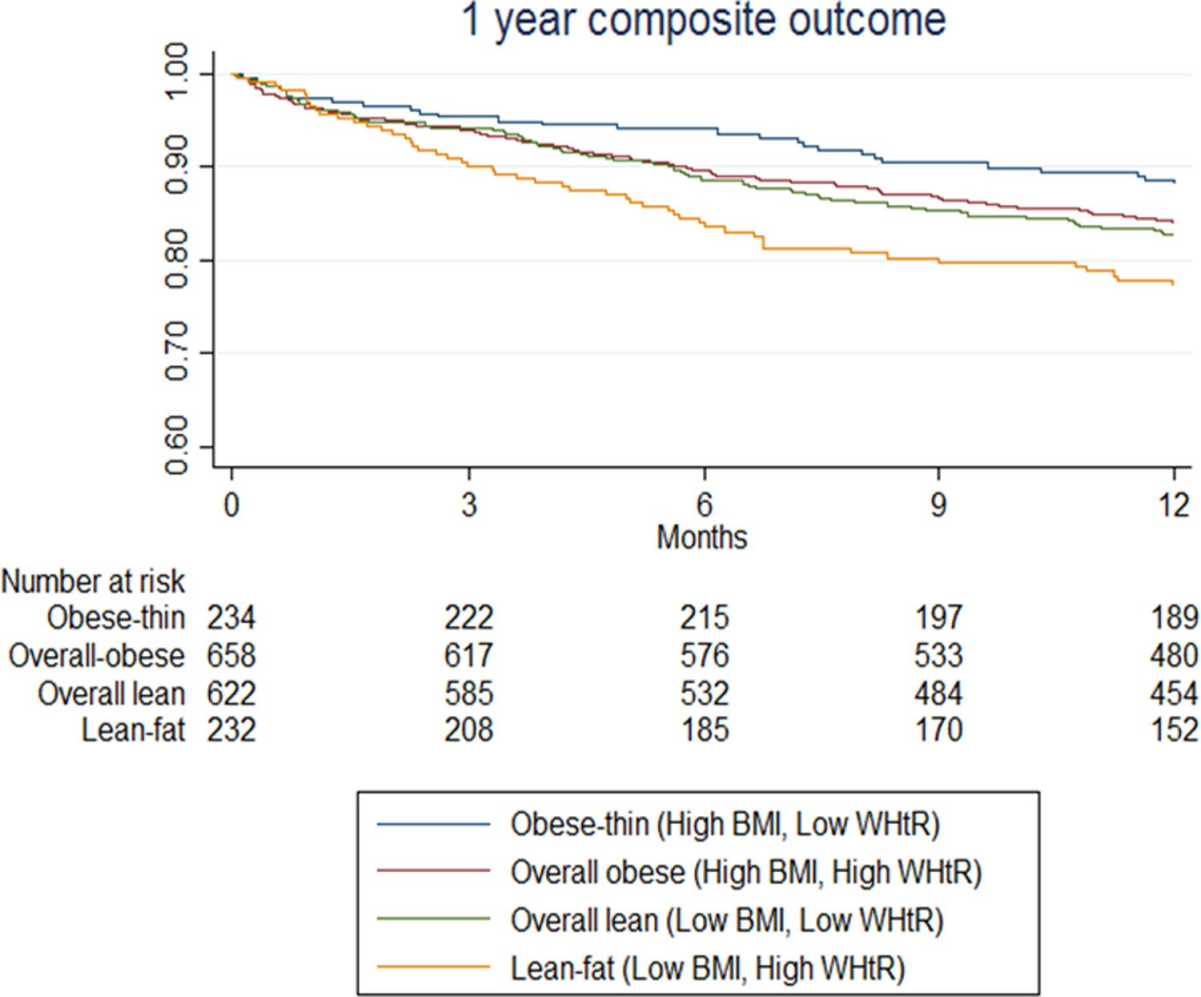
Kaplan–Meier survival curves of the composite outcome for combined BMI/WHtR groups. BMI, body mass index; WHtR, waist-to-height ratio.

Outcomes differed by BMI/WHtR groups. The risk of the composite outcome of death or HF hospitalisation was highest in the lean-fat (low BMI/high WHtR) group (51/232, 22.0%) and lowest in the obese-thin (high BMI/low WHtR) group (27/234, 11.5%). After adjustment for age, sex, HF subtype, NYHA class, systolic blood pressure, heart rate, comorbidities, and HF medications, the lean-fat (low BMI/high WHtR) group still had a higher risk of the composite outcome (adjusted hazard ratio 1.93, 95% CI 1.17–3.18, *p =* 0.010) (Table 2), compared to the obese-thin group. HF subtype (HFrEF or HFpEF) did not modify the association between the BMI/WHtR groups and the 1-year composite outcome (*p*_interaction_ = 0.355) (Fig 4).

Similar patterns were observed with 1-year all-cause mortality and HF hospitalisation. The mortality event rate (36/232, 15.5%) and adjusted risk of all-cause mortality were higher in the lean-fat (low BMI/high WHtR) group (adjusted hazard ratio 2.01, 95% CI 1.11–3.65, *p =* 0.022) than in the obese-thin group (Table 2). Results were similar in both HF subtypes (*p*_interaction_ = 0.609). Cardiovascular mortality was also higher in the lean-fat group (33/232, 14.5%) than in the obese-thin group (*p =* 0.026; Table 1). In sex-stratified analysis, higher WHtR (≥0.60) was associated with higher risk of death in both men and women, as observed in the overall cohort. However, our current study is underpowered to have such sex-specific conclusions statistically confirmed, as evident from the low number of events and large confidence intervals, especially in women (S7 Table).

With regards to QoL, the adjusted mean overall scores were not different between the 4 groups (*p =* 0.544). The lean-fat (low BMI/high WHtR) patients had lower adjusted mean social limitation scores compared to the obese-thin (high BMI/low WHtR) patients (61.9 versus 67.5, *p =* 0.034), while the difference in self-efficacy scores was attenuated after adjustment for demographic and clinical confounders (*p =* 0.510) (Table 3).

**Table 3:** Adjusted mean Kansas City Cardiomyopathy Questionnaire domain scores, by BMI/WHtR group.

Quality of life domains	Obese-thin (high BMI, low WHtR)	Overall obese (high BMI, high WHtR)	Overall lean (low BMI, low WHtR)	Lean-fat (low BMI, high WHtR)	*p*-Value
*n*	264	726	718	282	
Physical limitation score	69.0 (1.4)	71.0 (0.9)	71.2 (0.9)	69.3 (1.4)	0.396
Symptom stability score	62.8 (1.8)	60.7 (1.1)	61.5 (1.1)	64.6 (1.7)	0.241
Symptom frequency score	71.1 (1.5)	69.9 (0.9)	70.9 (0.9)	70.0 (1.4)	0.836
Symptom burden score	74.1 (1.5)	74.3 (0.9)	73.5 (0.9)	72.7 (1.4)	0.780
Total symptom score	72.6 (1.4)	72.1 (0.9)	72.2 (0.9)	71.4 (1.4)	0.934
Self-efficacy score	67.5 (1.6)	66.8 (1.0)	65.3 (1.0)	65.0 (1.5)	0.510
Social limitation score	67.5 (1.9)	65.1 (1.2)	63.1 (1.1)*	61.9 (1.9)*	0.102
Overall summary score	66.6 (1.2)	66.1 (0.8)	65.4 (0.7)	64.4 (1.2)	0.544
Clinical summary score	70.7 (1.2)	71.6 (0.7)	71.7 (0.7)	70.3 (1.2)	0.711

Data presented are adjusted mean scores (standard error of mean) (adjusted for age, sex, ethnicity, enrolment type, education, New York Heart Association class, systolic blood pressure, heart rate, heart failure subtype, history of coronary artery disease, atrial fibrillation, and diabetes).

*p < 0.05 compared against the obese-thin group.

BMI, body mass index; WHtR, waist-to-height ratio.

## Discussion

To our knowledge, this study provides the first prospective multinational data on the association of BMI versus WHtR with outcomes among Asian patients with HFpEF and HFrEF. We found a number of interesting results. First, BMI and abdominal obesity have directionally opposite relationships with outcomes—lower BMI, but higher WHtR, was associated with worse outcomes. Second, patients who were lean-fat (low BMI/high WHtR) were more likely to be women, to be from low-income countries or South/Southeast Asia, and to have the highest burden of diabetes, poorest QoL, and worst composite outcome. Third, results were consistent regardless of type of HF (HFpEF or HFrEF).

Previous reports on obesity and clinical outcomes in HF have predominantly relied on a single anthropometric measure of obesity (BMI or waist circumference or waist-to-hip ratio or WHtR) or examined these measurements in isolation from one another. Studies solely using BMI have consistently reported an obesity paradox, with overweight and obese patients with HF having better prognosis than their lean counterparts in both HFpEF and HFrEF [4–6,11]. However, more recent reports using measures of abdominal obesity have challenged this concept. Unlike for BMI, higher waist circumference was associated with higher risk of mortality across a wide range of left ventricular EF values [14], particularly HFpEF [13]. A combined measure of BMI and waist circumference showed higher risk of mortality in overall obese patients with high BMI and high waist circumference [19]. However, this was a single-centre study (*n =* 344) that included only patients with HFrEF. Our findings extend the prior literature by showing the association of combined measures of BMI/WHtR and outcomes in a large, well-characterised multinational Asian cohort of patients with HFrEF and HFpEF.

Our most striking finding is that BMI and WHtR have directionally contrasting relationships with clinical outcomes—higher BMI is associated with better, and higher WHtR with worse, outcomes. Despite lower BMI on average in Asian patients with HF compared to Western cohorts, we confirmed that the obesity paradox still applies to the Asian HF population when BMI is used to index obesity. This is consistent with limited previous reports from East Asia [7,8], but our data are the first to our knowledge available from wider geography including Southeast and South Asia. Higher metabolic reserve in face of the catabolic effects of HF is suggested as an explanation for the BMI paradox [10]. Although BMI is a convenient measure, it is known that it fails to distinguish between (1) components of body composition (fat mass, lean mass, skeletal muscle mass), (2) different body fat distributions (central versus peripheral adipose depots), and (3) fluid accumulation in decompensated HF and absolute weight gain [10].

In contrast to the findings for BMI, we observed that higher WHtR was associated with higher 1-year mortality and composite outcome, similar to some previous studies [13,14] but not others [19]. Consistent with our findings, higher waist circumference was associated with higher risk of all-cause mortality in the pure HFpEF cohort of TOPCAT, despite a strong paradoxical relationship between BMI and outcomes [13]. In the European BIOSTAT-CHF HF population [14], an association between higher waist-to-hip ratio and higher mortality was observed only in women, whereas we found similar associations in men and women in ASIAN-HF (i.e., sex did not modify the relationship between WHtR and outcomes in our study; *p*_interaction_ > 0.5). One other single-centre study, including only patients with HFrEF (*n =* 344) [19], reported contrary results in that HFrEF patients with normal BMI/normal waist circumference had the highest hazards of 2-year mortality compared to those with high BMI/high waist circumference—discordant results that may be due to exclusion of lean patients (with BMI < 18.5 kg/m^2^) and patients with HFpEF, in whom prevalence of central obesity is higher. Importantly, our study is the first demonstration to our knowledge of the impact of WHtR, an index of central obesity, on HF outcomes in Asia. While there was greater prevalence of central obesity among patients with HFpEF than HFrEF, it was equally associated with worse outcomes in both HF subtypes. Indeed, abdominal measures of adiposity (waist circumference, waist-to-hip ratio, WHtR) more accurately reflect body composition than BMI [20]. Mechanistic insights into abdominal adiposopathy include suggestions of a pro-inflammatory pathology. Adipose tissues actively produce inflammatory cytokines, potentially leading to diastolic dysfunction [21]. Experimental models have shown strong relationships between visceral obesity and increased cardiac macrophage infiltration and cytokine gene expression, exacerbating myocardial hypertrophy, fibrosis, and injury [22].

Our findings highlight a particularly susceptible group of lean-fat (low BMI/high WHtR) patients who were more likely to be women, with a higher prevalence of diabetes, poorer QoL scores, and worse composite outcome, compared to all other groups. Central adiposity is associated with cardiac dysfunction, independent of BMI, even in lean individuals [23]. The thrifty gene hypothesis, maternal insufficiency, foetal undernutrition, and differences in body fat distribution have been suggested to contribute to the higher prevalence of lean diabetes in Asia [24,25]. Our preliminary evidence from body composition analysis showing that higher waist-to-hip ratio was independently linked with higher trunk fat and lower skeletal muscle mass suggests the presence of sarcopenia in the lean-fat patients. Whether sarcopenia may be a treatable target in HF remains to be proven. Interestingly, the commonly observed BMI paradox is obliterated when cardiorespiratory fitness is increased/preserved [26], presumably due to preservation of skeletal muscle mass. It is also noteworthy that most of the lean-fat patients in our study were from South Asia and Southeast Asia, while patients from Northeast Asia were more likely to be overall lean. Indeed, ethnic differences in body fat distribution have been reported previously in the INSPIRE ME IAA study, which included white, black, Hispanic, East Asian, and South Asian individuals. CT-scan assessment revealed that East Asians, followed by South Asians, had a more detrimental abdominal fat deposition, despite low BMI [27].

Asians exhibit remarkable variability in body fat distribution for a given BMI, and viscerally obese patients with HF have the worst outcomes. Information from a single anthropometric measure in isolation fails to provide the necessary insights into body fat distribution. More accurate estimators of visceral fat, such as computer tomography scans, magnetic resonance imaging, and dual energy X-ray absorptiometry, are cost intensive or involve the risk of radiation [10]. Whilst not perfect, a combination measure of BMI and WHtR is a simple and convenient alternative for estimation of body fat distribution. Targeting visceral obesity with inhibitors of aldosterone, neprilysin, and, particularly, sodium glucose transporter 2 (SGLT2) have been suggested for management of obesity-related HF, pending confirmation from ongoing large-scale trials [28].

### Strengths and limitations

Selection bias and residual confounding are inevitable in such multinational observational studies. Lack of repeat assessments of BMI or waist measures precludes any inferences involving loss of muscle mass in the progression of HF. Unmeasured factors such as differences in healthcare system, economic development, and nutritional status across the regions are potential confounders. Patients with body composition analysis are more recent recruits who do not have follow-up data available for outcome analysis. Key strengths of this study include the prospective design, that the study spans across widespread geography in Asia (46 centres from 11 regions), uniform data collection, meticulous follow-up, and independent adjudication of outcomes. We utilised abdominal measures and body composition data in addition to BMI to investigate the obesity paradox in both HFpEF and HFrEF in Asia.

### Implications and next steps

Obesity in HF poses formidable public health challenges for health policymakers, health service providers, and patient caregivers in many low- and middle-income countries. The controversy surrounding the obesity paradox makes it even more challenging for clinicians to manage concomitant HF and obesity. Combined usage of BMI and abdominal measures could potentially inform HF management better, especially among the particularly vulnerable patients with low BMI and high WHtR. Clear national policies that underscore prevention of abdominal obesity and promotion of a healthy BMI, through awareness, education, and lifestyle modification, need to be championed.

## Conclusion

Among Asian patients with HF, we observed that higher BMI was associated with better outcomes whereas higher WHtR conferred worse outcomes. Our findings highlight a particularly susceptible group of lean-fat (low BMI/high WHtR) patients who were more likely to be women, with a higher prevalence of diabetes, poorer QoL scores, and worse composite outcome, compared to all other groups.

## Supporting information

S1 Checklist STROBE checklist. (DOCX)

S1 Table List of ethics committees involved in ASIAN-HF registry. (DOCX)

S2 Table Baseline characteristics by BMI strata for entire cohort (*n =* 5,964). (DOCX)

S3 Table Baseline characteristic comparison of patients with (*n =* 2,051) and without (*n =* 3,913) waist circumference measurements. (DOCX)

S4 Table Baseline characteristics by BMI and waist-to-height ratio quartiles (*n =* 2,051). (DOCX)

S5 Table Baseline characteristics of patient subset with body composition analysis (*n =* 311). (DOCX)

S6 Table Association of BMI with outcomes in the entire cohort (*n =* 5,395). (DOCX)

S7 Table Sex-stratified analysis of waist-to-height ratio quartiles and outcomes. (DOCX)

S1 Text ASIAN-HF executive committee and investigators. (DOCX)

## Abbreviations

ASIAN-HF: Asian Sudden Cardiac Death in Heart Failure
BMI: body mass index
EF: ejection fraction
eGFR: estimated glomerular filtration rate
HF: heart failure
HFpEF: heart failure with preserved ejection fraction
HFrEF: heart failure with reduced ejection fraction
KCCQ: Kansas City Cardiomyopathy Questionnaire
NYHA: New York Heart Association
QoL: quality of life
WHtR: waist-to-height ratio

## References

[1] World Health Organization. Obesity and overweight. Geneva: World Health Organization; 2018 [cited 2019 Aug 27]. Available from: https://www.who.int/news-room/fact-sheets/detail/obesity-and-overweight.

[2] Lam CS, Teng T-HK, Tay WT, Anand I, Zhang S, Shimizu W, et al Regional and ethnic differences among patients with heart failure in Asia: the Asian sudden cardiac death in heart failure registry. Eur Heart J. 2016;37(41):3141–53. 27502121 10.1093/eurheartj/ehw331

[3] WHO Expert Consultation. Appropriate body-mass index for Asian populations and its implications for policy and intervention strategies. Lancet. 2004;363(9403):157–63. 14726171 10.1016/S0140-6736(03)15268-3

[4] Haass M, Kitzman DW, Anand IS, Miller A, Zile MR, Massie BM, et al Body mass index and adverse cardiovascular outcomes in heart failure patients with preserved ejection fraction: results from the Irbesartan in Heart Failure with Preserved Ejection Fraction (I-PRESERVE) trial. Circ Heart Fail. 2011;4(3):324–31. 10.1161/CIRCHEARTFAILURE.110.959890 21350053 PMC3100162

[5] Kenchaiah S, Pocock SJ, Wang D, Finn PV, Zornoff LA, Skali H, et al Body mass index and prognosis in patients with chronic heart failure: insights from the Candesartan in Heart failure: Assessment of Reduction in Mortality and morbidity (CHARM) program. Circulation. 2007;116(6):627–36. 10.1161/circulationaha.106.679779 17638930

[6] Padwal R, McAlister FA, McMurray JJ, Cowie MR, Rich M, Pocock S, et al The obesity paradox in heart failure patients with preserved versus reduced ejection fraction: a meta-analysis of individual patient data. Int J Obes (Lond). 2014;38(8):1110–4. 10.1038/ijo.2013.203 24173404

[7] Hamaguchi S, Tsuchihashi-Makaya M, Kinugawa S, Goto D, Yokota T, Goto K, et al Body mass index is an independent predictor of long-term outcomes in patients hospitalized with heart failure in Japan. Circ J. 2010;74(12):2605–11. 21060207 10.1253/circj.cj-10-0599

[8] Lin GM, Li YH, Yin WH, Wu YW, Chu PH, Wu CC, et al The obesity-mortality paradox in patients with heart failure in Taiwan and a collaborative meta-analysis for East Asian patients. Am J Cardiol. 2016;118(7):1011–8. 10.1016/j.amjcard.2016.06.056 27521221

[9] Tromp J, Tay WT, Ouwerkerk W, Teng TK, Yap J, MacDonald MR, et al Multimorbidity in patients with heart failure from 11 Asian regions: a prospective cohort study using the ASIAN-HF registry. PLoS Med. 2018;15(3):e1002541 10.1371/journal.pmed.1002541 29584721 PMC5870945

[10] Neeland IJ, Poirier P, Despres JP. Cardiovascular and metabolic heterogeneity of obesity: clinical challenges and implications for management. Circulation. 2018;137(13):1391–406. 10.1161/CIRCULATIONAHA.117.029617 29581366 PMC5875734

[11] Iliodromiti S, Celis-Morales CA, Lyall DM, Anderson J, Gray SR, Mackay DF, et al The impact of confounding on the associations of different adiposity measures with the incidence of cardiovascular disease: a cohort study of 296 535 adults of white European descent. Eur Heart J. 2018;39(17):1514–20. 10.1093/eurheartj/ehy057 29718151 PMC5930252

[12] Myint PK, Kwok CS, Luben RN, Wareham NJ, Khaw KT. Body fat percentage, body mass index and waist-to-hip ratio as predictors of mortality and cardiovascular disease. Heart. 2014;100(20):1613–9. 10.1136/heartjnl-2014-305816 24966306

[13] Tsujimoto T, Kajio H. Abdominal obesity is associated with an increased risk of all-cause mortality in patients with HFpEF. J Am Coll Cardiol. 2017;70(22):2739–49. 10.1016/j.jacc.2017.09.1111 29191321

[14] Streng KW, Voors AA, Hillege HL, Anker SD, Cleland JG, Dickstein K, et al Waist-to-hip ratio and mortality in heart failure. Eur J Heart Fail. 2018;20(9):1269–77. 10.1002/ejhf.1244 29963737

[15] Lam CS, Anand I, Zhang S, Shimizu W, Narasimhan C, Park SW, et al Asian Sudden Cardiac Death in Heart Failure (ASIAN-HF) registry. Eur J Heart Fail. 2013;15(8):928–36. 10.1093/eurjhf/hft045 23568645

[16] Chia YMF, Teng T-HK, Tan ES, Tay WT, Richards AM, Chin CWL, et al Disparity between indications for and utilization of implantable cardioverter defibrillators in Asian patients with heart failure. Circ Cardiovasc Qual Outcomes. 2017;10(11):e003651 10.1161/CIRCOUTCOMES.116.003651 29150533

[17] Luo N, Teng TK, Tay WT, Anand IS, Kraus WE, Liew HB, et al Multinational and multiethnic variations in health-related quality of life in patients with chronic heart failure. Am Heart J. 2017;191:75–81. 10.1016/j.ahj.2017.06.016 28888273 PMC5663287

[18] Bedogni G, Malavolti M, Severi S, Poli M, Mussi C, Fantuzzi AL, et al Accuracy of an eight-point tactile-electrode impedance method in the assessment of total body water. Eur J Clin Nutr. 2002;56(11):1143–8. 10.1038/sj.ejcn.1601466 12428182

[19] Clark AL, Fonarow GC, Horwich TB. Waist circumference, body mass index, and survival in systolic heart failure: the obesity paradox revisited. J Card Fail. 2011;17(5):374–80. 10.1016/j.cardfail.2011.01.009 21549293

[20] Cornier MA, Despres JP, Davis N, Grossniklaus DA, Klein S, Lamarche B, et al Assessing adiposity: a scientific statement from the American Heart Association. Circulation. 2011;124(18):1996–2019. 10.1161/CIR.0b013e318233bc6a 21947291

[21] Ballak DB, Stienstra R, Tack CJ, Dinarello CA, van Diepen JA. IL-1 family members in the pathogenesis and treatment of metabolic disease: focus on adipose tissue inflammation and insulin resistance. Cytokine. 2015;75(2):280–90. 10.1016/j.cyto.2015.05.005 26194067 PMC4553099

[22] Murase T, Hattori T, Ohtake M, Abe M, Amakusa Y, Takatsu M, et al Cardiac remodeling and diastolic dysfunction in DahlS.Z-Lepr(fa)/Lepr(fa) rats: a new animal model of metabolic syndrome. Hypertens Res. 2012;35(2):186–93. 10.1038/hr.2011.157 21918527

[23] Selvaraj S, Martinez EE, Aguilar FG, Kim K-YA, Peng J, Sha J, et al Association of central adiposity with adverse cardiac mechanics: findings from the hypertension genetic epidemiology network study. Circ Cardiovasc Imaging. 2016;9(6):e004396 10.1161/CIRCIMAGING.115.004396 27307550 PMC4911824

[24] Gujral UP, Weber MB, Staimez LR, Narayan KMV. Diabetes among non-overweight individuals: an emerging public health challenge. Curr Diab Rep. 2018;18(8):60 10.1007/s11892-018-1017-1 29974263

[25] Yajnik CS, Yudkin JS. The Y-Y paradox. Lancet. 2004;363(9403):163 10.1016/s0140-6736(03)15269-5 14726172

[26] Carbone S, Popovic D, Lavie CJ, Arena R. Obesity, body composition and cardiorespiratory fitness in heart failure with preserved ejection fraction. Future Cardiol. 2017 8 10 10.2217/fca-2017-0023 28795590

[27] Nazare JA, Smith JD, Borel AL, Haffner SM, Balkau B, Ross R, et al Ethnic influences on the relations between abdominal subcutaneous and visceral adiposity, liver fat, and cardiometabolic risk profile: the International Study of Prediction of Intra-Abdominal Adiposity and Its Relationship With Cardiometabolic Risk/Intra-Abdominal Adiposity. Am J Clin Nutr. 2012;96(4):714–26. 10.3945/ajcn.112.035758 22932278

[28] Packer M, Kitzman DW. Obesity-related heart failure with a preserved ejection fraction: the mechanistic rationale for combining inhibitors of aldosterone, neprilysin, and sodium-glucose cotransporter-2. JACC Heart Fail. 2018;6(8):633–9. 10.1016/j.jchf.2018.01.009 29525327

