# Supplementary material for: Association of obesity with heart failure outcomes in 11 Asian regions: A cohort study
Source: PLoS Med. 2019 Sep 24;16(9):e1002916. doi: 10.1371/journal.pmed.1002916 (PMC6759142; doi:10.1371/journal.pmed.1002916)
Supplement: S1 Table — (DOCX) [file pmed.1002916.s002.docx]

**S1 Table. List of ethics committees involved in ASIAN-HF Registry**

| **Country** | **Site ID** | **Name of Ethics Committee involved for local submissions on ASIAN-HF Registry** |
| --- | --- | --- |
| China | 0101 | Ethics Committee of Fuwai Hospital |
| China | 102 | Ethics Committee of Zhongshan Hospital of Fudan University |
| China | 103 | Ethics Committee of Ruijin Hospital, Shanghai Jiaotong University School of Medicine |
| China | 105 | Ethics Committee of Jiangsu Province Hospital |
| Hong Kong | 201 | Joint Chinese University of Hong Kong – New Territories East Cluster Chinese Research Ethics Committee |
| India | 301 | Medanta (Independent Ethics Committee) |
| India | 302 | CARE Foundation Institutional Ethical Committee |
| India | 303 | Ethics Committee of Care Institute of Medical Sciences |
| India | 304 | Drug Trial Ethics Committee (DTEC) |
| India | 306 | Institutional Ethical Committee Westfort Hi-tech Hospital Ltd |
| India | 307 | Ethics Committee Sir Ganga Ram Hospital |
| Indonesia | 401 | Komite Etik Rumah Sakit Jantung Harapan Kita |
| Indonesia | 402 | Komite Etik Penelitian Kesehatan |
|  |  | Fakultas Kedokteran Universitas Padjadjaran |
| Indonesia | 403 and 404 | Komite EtikFakultas Kedokteran Universitas Indonesia |
| Japan | 501 | Research Ethics Committee of National Cerebral and Cardiovascular Center |
| Japan | 502 | Independent Ethics Committee of Kinki University Hospital |
| Japan | 503 | Hospital Ethics Committee of Department of Cardiology, Tokyo Women's Medical University |
| Japan | 504 | Ethics Committee of Toho University Omori Medical Center |
| Japan | 505 | Independent Ethics Committee of Nippon Medical School Hospital |
| Malaysia | 601 | IJN |
| Malaysia | 602 | UMMC |
| Malaysia | 603 | MREC |
| Malaysia | 604 | MREC |
| Philippines | 701 | Manila Doctors Hospital Institutional Review Board |
| Philippines | 702 | Makati Medical Center Institutional Review Board |
| Philippines | 703 | Philippine Heart Center Institutional Ethics Review Board |
| Singapore | 801 | DSRB |
| Singapore | 802 | CIRB |
| Singapore | 803 | DSRB |
| Singapore | 804 | CIRB |
| Singapore | 805 | DSRB |
| Singapore | 806 | CIRB |
| South Korea | 901 | IRB of Korea University Anam Hospital |
| South Korea | 902 | IRB of Korea University Guro Hospital |
| South Korea | 903 | IRB of Hyewon Medical Foundation SeJong General Hospital |
| South Korea | 904 | IRB of Korea University Ansan Hospital |
| South Korea | 905 | IRB of Severance Hospital, Yonsei University Health System |
| South Korea | 906 | IRB of Chonnam National University Hospital |
| Thailand | 1001 | Ethical Clearance Committee on Human Rights Related to Research Involving Human Subjects Faculty of Medicine Ramathibodi Hospital, Mahidol University |
| Thailand | 1002 | The Institutional Review Board, Royal Thai Army Medical Department |
| Thailand | 1003 | Research Ethics Committee Faculty of Medicine Chiang Mai University |
| Taiwan | 1101 | Mackay Memorial Hospital Institutional Review Board |
| Taiwan | 1102 | Taiwan University Hospital Research Ethics Committee |
| Taiwan | 1103 | Taipei Veterans General Hospital Institutional Review Board |
| Taiwan | 1104 | China Medical University Hospital Research Ethics Committee |
