# Supplementary material for: Association of obesity with heart failure outcomes in 11 Asian regions: A cohort study
Source: PLoS Med. 2019 Sep 24;16(9):e1002916. doi: 10.1371/journal.pmed.1002916 (PMC6759142; doi:10.1371/journal.pmed.1002916)
Supplement: S2 Table — (DOCX) [file pmed.1002916.s003.docx]

| **S2 Table. Baseline characteristics by BMI strata for entire cohort (n=5,964)** | | | | | |  |  |
| --- | --- | --- | --- | --- | --- | --- | --- |
|  |  | |  | | | | |
|  | **BMI groups, kg/m^2^** | | | | |  |  |
|  | **<18.5** | **18.5-<23** | | **23-<27.5** | **>=27.5** | **P-value (ANOVA/ χ^2^)** | **P for linear trend** |
| **Demographics & clinical characteristics** | |  | |  |  |  |  |
| n | 358 | 1759 | | 2264 | 1583 |  |  |
| HFpEF | 29 (8.1) | 204 (11.6) | | 339 15.0) | 380 (24.0) | <0.001 | <0.001 |
| Age, years | 64.0 (15.5) | 64.0 (13.2) | | 61.3 (12.6) | 57.8 (13.0) | <0.001 | <0.001 |
| Women | 134 (37.4) | 449 (25.5) | | 532 (23.5) | 456 (28.8) | <0.001 | 0.386 |
| NYHA |  |  | |  |  | 0.067 | 0.017 |
| Class I or II | 212 (63.5) | 1046 (65.1) | | 1420 (68.3) | 984 (68.2) |  |  |
| Class III or IV | 122 (36.5) | 562 (35) | | 660 (31.7) | 458 (31.8) |  |  |
| Systolic blood pressure, mmHg | 112.6 (20.8) | 117.1 (20.5) | | 120.9 (20.4) | 125.4 (21.1) | <0.001 | <0.001 |
| Diastolic blood pressure, mmHg | 67.4 (11.7) | 70.1 (12.3) | | 72.6 (12.2) | 75.3 (12.8) | <0.001 | <0.001 |
| Heart rate, bpm | 79.1 (16.3) | 77.7 (15.3) | | 79.0 (16.2) | 79.7 (15.4) | 0.003 | 0.004 |
| Waist circumference, cm (n=2051) | 76.9 (10.7) | 84.5 (9.0) | | 91.6 (8.8) | 102 .6 (12.1) | <0.001 | <0.001 |
| eGFR, mL/min/1.73 m2 | 67.0 (30.6) | 65.2 (26.8) | | 64.6 (28.1) | 65.1 (28.4) | 0.569 | 0.601 |
|  |  |  | |  |  |  |  |
| **Comorbidities** |  |  | |  |  |  |  |
| Ischemic HF | 148 (41.5) | 784 (44.7) | | 1045 (46.2) | 677 (42.8) | 0.122 | 0.835 |
| Hypertension | 144 (40.3) | 833 (47.5) | | 1262 (55.8) | 1018 (64.5) | <0.001 | <0.001 |
| Diabetes | 86 (24.1) | 632 (36.0) | | 954 (42.2) | 783 (49.6) | <0.001 | <0.001 |
| Smoking, ever | 142 (40.0) | 768 (43.8) | | 965 (42.7) | 632 (40.0) | 0.126 | 0.164 |
| Alcohol, ever | 66 (18.6) | 525 (29.9) | | 626 (27.7) | 407 (25.8) | <0.001 | 0.795 |
|  |  |  | |  |  |  |  |
| **Outcomes at 1year (n=5,397)** |  |  | |  |  |  |  |
| All-cause mortality | 54 (16.9) | 177 (11.3) | | 196 (9.5) | 98 (6.7) | <0.001 | <0.001 |
| Cardiovascular mortality | 44 (14.2) | 151 (9.8) | | 164 (8.1) | 85 (5.9) | <0.001 | <0.001 |
| Composite outcome | 86 (27.0) | 349 (22.3) | | 377 (18.4) | 258 (17.7) | <0.001 | <0.001 |
|  |  |  | |  |  |  |  |
