# Supplementary material for: Association of obesity with heart failure outcomes in 11 Asian regions: A cohort study
Source: PLoS Med. 2019 Sep 24;16(9):e1002916. doi: 10.1371/journal.pmed.1002916 (PMC6759142; doi:10.1371/journal.pmed.1002916)
Supplement: S3 Table — (DOCX) [file pmed.1002916.s004.docx]

**S3 Table. Baseline characteristics comparison of patients with (n=2,051) and without (n=3,913) waist circumference (WC) measurements**

|  | **Total** | **With WC measure** | **Without WC measure** | **p-value** |
| --- | --- | --- | --- | --- |
| n | 5964 | 2051 | 3913 |  |
| HFpEF | 952 (16) | 139 (6.8) | 813 (20.8) | <0.001 |
| Age, years | 61.3 (13.3) | 60.8 (12.9) | 61.6 (13.5) | 0.018 |
| Women | 1571 (26.3) | 493 (24) | 1078 (27.5) | 0.003 |
| NYHA |  |  |  | 0.078 |
| Class I or II | 3662 (67) | 1316 (68.5) | 2346 (66.2) |  |
| Class III or IV | 1802 (33) | 604 (31.5) | 1198 (33.8) |  |
| Systolic blood pressure, mmHg | 120.5 (21) | 119.3 (19.7) | 121.1 (21.6) | 0.001 |
| Diastolic blood pressure, mmHg | 72.3 (12.6) | 72.6 (11.8) | 72.1 (13) | 0.109 |
| Heart rate, bpm | 78.8 (15.8) | 78.8 (14.8) | 78.8 (16.2) | 0.866 |
| Body mass index, kg/m^2^ | 25.3 (5.3) | 25 (5.2) | 25.4 (5.4) | 0.009 |
| eGFR, mL/min/1.73 m^2^ | 65.1 (28) | 65.4 (27.7) | 64.9 (28.1) | 0.53 |
