# Supplementary material for: Association of obesity with heart failure outcomes in 11 Asian regions: A cohort study
Source: PLoS Med. 2019 Sep 24;16(9):e1002916. doi: 10.1371/journal.pmed.1002916 (PMC6759142; doi:10.1371/journal.pmed.1002916)
Supplement: S4 Table — (DOCX) [file pmed.1002916.s005.docx]

|  | **S4 Table. Baseline characteristics by BMI and waist-to-height ratio quartiles (n=2,051)** | | | | | | | | | | | | | | | | |  |  |
| --- | --- | --- | --- | --- | --- | --- | --- | --- | --- | --- | --- | --- | --- | --- | --- | --- | --- | --- | --- |
|  | |  |  |  |  |  |  | |  | |  |  | | | | | | |  |
|  | | **BMI quartiles, kg/m^2^** | | | | | |  | | **Waist-to-height ratio quartiles** | | | | | | | |  |  |
|  | | **<21.8** | **21.8-<24.5** | **24.5-<27.8** | **≥27.8** | **P-value (ANOVA/ χ^2^)** | **P for linear trend** | |  | | **<0.51** | | **0.51-<0.55** | **0.55-<0.60** | **≥0.60** | **P-value (ANOVA/ χ^2^)** | **P for linear trend** |  |  |
| **Demographics & Clinical characteristics** | | |  |  |  |  |  | |  | |  | |  |  |  |  |  |  |  |
| n | | 517 | 574 | 491 | 469 |  |  | |  | | 512 | | 514 | 512 | 515 |  |  |  |  |
| HFpEF | | 17 (3.3) | 24 (4.2) | 35 (7.1) | 63 (13.4) | <0.001 | <0.001 | |  | | 21 (4.1) | | 15 (2.9) | 27 (5.3) | 76 (14.8) | <0.001 | <0.001 |  |  |
| Age, years | | 63.7 (13.6) | 60.5 (12.2) | 60.7 (12.3) | 57.9 (13) | <0.001 | <0.001 | |  | | 60.2 (13.6) | | 61.5 (12.8) | 60.1 (12.2) | 61.4 (12.9) | 0.160 | 0.374 |  |  |
| Women | | 141 (27.3) | 115 (20) | 107 (21.8) | 130 (27.7) | 0.005 | 0.75 | |  | | 108 (21.1) | | 77 (15) | 131 (25.5) | 177 (34.5) | <0.001 | <0.001 |  |  |
| NYHA | |  |  |  |  | 0.225 | 0.522 | |  | |  | |  |  |  | 0.643 | 0.752 |  |  |
| Class I or II | | 344 (69.8) | 347 (64.9) | 320 (70) | 305 (70.1) |  |  | |  | | 327 (67.8) | | 321 (67.6) | 342 (71.3) | 328 (67.6) |  |  |  |  |
| Class III or IV | | 149 (30.2) | 188 (35.1) | 137 (30) | 130 (29.9) |  |  | |  | | 155 (32.2) | | 154 (32.4) | 138 (28.7) | 157 (32.4) |  |  |  |  |
| Systolic BP, mmHg | | 114.8 (19.9) | 117.9 (18.1) | 120.5 (20.0) | 124.5 (19.7) | <0.001 | <0.001 | |  | | 115.7 (19.8) | | 117.2 (18.1) | 120.9 (19.6) | 123.2 (20.4) | <0.001 | <0.001 |  |  |
| Diastolic BP, mmHg | | 70.1 (11.2) | 72.3 (11.3) | 72.9 (12.5) | 75.5 (11.6) | <0.001 | <0.001 | |  | | 72.0 (11.2) | | 71.9 (11.7) | 73.3 (11.7) | 73.4 (12.3) | 0.072 | 0.018 |  |  |
| Heart rate, bpm | | 78.1 (14.1) | 79.1 (15.1) | 78.6 (14.8) | 79.6 (15.2) | 0.421 | 0.205 | |  | | 78.7 (15.6) | | 79 (14.9) | 79.5 (14.6) | 78.2 (14.0) | 0.504 | 0.769 |  |  |
| BMI, kg/m^2^ | | 19.6 (1.7) | 23.1 (0.8) | 26.1 (0.9) | 32.2 (5.1) | <0.001 | <0.001 | |  | | 21.2 (3.0) | | 23.9 (3.2) | 25.4 (4.1) | 29.6 (5.9) | <0.001 | <0.001 |  |  |
| eGFR, mL/min/1.73 m^2^ | | 64.2 (27.2) | 66.3 (27.6) | 64.7 (27.9) | 66.4 (28.4) | 0.605 | 0.441 | |  | | 66.9 (26.3) | | 64.6 (27.5) | 66.7 (28.0) | 63.7 (28.9) | 0.332 | 0.233 |  |  |
| **Comorbidities** | |  |  |  |  |  |  | |  | |  | |  |  |  |  |  |  |  |
| Ischemic HF | | 255 (49.4) | 282 (49.2) | 246 (50.1) | 217 (46.3) | 0.66 | 0.39 | |  | | 244 (47.7) | | 263 (51.2) | 255 (49.7) | 239 (46.6) | 0.533 | 0.617 |  |  |
| Hypertension | | 234 (45.3) | 280 (48.9) | 287 (58.5) | 280 (60) | <0.001 | <0.001 | |  | | 226 (44.2) | | 251 (48.9) | 278 (54.3) | 328 (63.9) | <0.001 | <0.001 |  |  |
| Coronary artery disease | | 259 (50.2) | 311 (54.3) | 263 (53.6) | 228 (48.8) | 0.239 | 0.654 | |  | | 254 (49.7) | | 279 (54.4) | 273 (53.3) | 256 (49.9) | 0.367 | 0.959 |  |  |
| Diabetes | | 169 (32.8) | 223 (38.9) | 214 (43.6) | 220 (47.1) | <0.001 | <0.001 | |  | | 151 (29.5) | | 208 (40.5) | 228 (44.5) | 241 (47) | <0.001 | <0.001 |  |  |
| Prior stroke | | 22 (4.3) | 42 (7.3) | 30 (6.1) | 38 (8.1) | 0.066 | 0.038 | |  | | 26 (5.1) | | 26 (5.1) | 29 (5.7) | 52 (10.1) | 0.002 | 0.001 |  |  |
| Smoking, ever | | 232 (45) | 256 (44.7) | 221 (45) | 199 (42.6) | 0.856 | 0.495 | |  | | 248 (48.6) | | 233 (45.4) | 225 (43.9) | 203 (39.6) | 0.027 | 0.004 |  |  |
| Alcohol, ever | | 129 (25) | 179 (31.2) | 135 (27.5) | 132 (28.3) | 0.164 | 0.523 | |  | | 156 (30.6) | | 137 (26.7) | 155 (30.3) | 128 (25) | 0.153 | 0.134 |  |  |
| **Outcomes at 1year (n=1,746)** | | | | | | | | | | | | | | | | | |  | |
| All-cause mortality | | 59 (13.8) | 55 (11.3) | 47 (11.2) | 30 (7.3) | 0.026 | 0.004 | |  | | 47 (11.1) | | 46 (10.5) | 43 (9.9) | 55 (12.1) | 0.744 | 0.736 |  |  |
| Cardiovascular mortality | | 49 (11.5) | 49 (10) | 42 (10) | 24 (5.8) | 0.031 | 0.008 | |  | | 39 (9.2) | | 41 (9.3) | 39 (9.0) | 45 (9.9) | 0.959 | 0.968 |  |  |
| Composite outcome | | 83 (19.4) | 79 (16.2) | 63 (15) | 61 (14.8) | 0.232 | 0.063 | |  | | 62 (14.7) | | 69 (15.7) | 63 (14.6) | 91 (20.1) | 0.094 | 0.091 |  |  |
