# Supplementary material for: Association of obesity with heart failure outcomes in 11 Asian regions: A cohort study
Source: PLoS Med. 2019 Sep 24;16(9):e1002916. doi: 10.1371/journal.pmed.1002916 (PMC6759142; doi:10.1371/journal.pmed.1002916)
Supplement: S5 Table — (DOCX) [file pmed.1002916.s006.docx]

| **S5 Table. Baseline characteristics of patient subset with body composition analysis (n=311)** | | | | |  |  |
| --- | --- | --- | --- | --- | --- | --- |
|  |  |  |  |  |  |  |
| **Baseline characteristic** | **Patient data available** | **Obese-thin** | **Overall obese** | **Overall lean** | **Lean-fat** | **p-value** |
| n | 311 | 43 | 113 | 103 | 52 |  |
| Age, years | 280 | 56.8 (12.4) | 53.4 (11.0) | 60.5 (9.8) | 56.9 (9.3) | <0.001 |
| Men | 287 | 23 (62.2%) | 88 (82.2%) | 73 (77.7%) | 45 (91.8%) | 0.007 |
| HFpEF | 275 | 10 (27.0%) | 21 (20.6%) | 12 (13.5%) | 7 (14.9%) | 0.26 |
| NYHA class I or II | 274 | 32 (88.9%) | 98 (95.1%) | 84 (94.4%) | 44 (95.7%) | 0.42 |
| Systolic blood pressure, mmHg | 273 | 135.8 (25.8) | 129.0 (19.1) | 124.9 (21.7) | 128.9 (20.9) | 0.079 |
| Diastolic blood pressure, mmHg | 273 | 72.6 (15.8) | 75.7 (13.9) | 69.9 (12.9) | 75.1 (13.1) | 0.026 |
| Heart rate, bpm | 272 | 75.2 (15.4) | 76.3 (13.9) | 73.2 (12.7) | 73.2 (13.6) | 0.4 |
| Serum creatinine, umol/L | 242 | 118.8 (42.6) | 112.1 (47.8) | 107.5 (41.0) | 109.1 (80.7) | 0.76 |
| Ischaemic aetiology of HF | 268 | 21 (61.8%) | 43 (42.2%) | 50 (58.1%) | 27 (58.7%) | 0.17 |
| Coronary artery disease | 268 | 18 (52.9%) | 49 (49%) | 56 (64%) | 32 (68%) | 0.244 |
| Atrial fibrillation | 272 | 5 (14.3%) | 20 (19.8%) | 19 (21.3%) | 8 (17.0%) | 0.8 |
| Hypertension | 272 | 25 (71.4%) | 71 (70.3%) | 51 (57.3%) | 30 (63.8%) | 0.24 |
| Diabetes | 272 | 26 (76.5%) | 55 (53.9%) | 46 (51.7%) | 23 (48.9%) | 0.17 |
| BMI, kg/m^2^ | 311 | 31.6 (4.0) | 33.1 (13.3) | 22.2 (2.2) | 24.3 (2.5) | <0.001 |
| Waist-to-hip ratio | 311 | 0.78 (0.10) | 1.00 (0.06) | 0.82 (0.07) | 2.14 (8.61) | 0.13 |
| Waist-to-height ratio | 91 | 0.67 (0.09) | 0.66 (0.10) | 0.52 (0.05) | 0.54 (0.04) | <0.001 |
| Weight, kg | 311 | 83.2 (16.8) | 88.7 (14.8) | 59.5 (8.1) | 67.4 (8.8) | <0.001 |
| Fat mass, % | 311 | 35.4 (9.9) | 39.7 (6.6) | 25.9 (9.6) | 32.8 (8.2) | <0.001 |
| Skeletal muscle, % | 311 | 35.4 (5.6) | 33.3 (4.0) | 39.8 (5.7) | 37.8 (6.1) | <0.001 |
| Trunk fat, % | 311 | 13.0 (3.7) | 17.9 (3.7) | 7.2 (2.6) | 11.5 (2.1) | <0.001 |
| Fat-to-skeletal ratio | 311 | 1.07 (0.45) | 1.24 (0.37) | 0.71 (0.47) | 0.88 (0.22) | <0.001 |
|  |  |  |  |  |  |  |
