# Supplementary material for: Association of obesity with heart failure outcomes in 11 Asian regions: A cohort study
Source: PLoS Med. 2019 Sep 24;16(9):e1002916. doi: 10.1371/journal.pmed.1002916 (PMC6759142; doi:10.1371/journal.pmed.1002916)
Supplement: S6 Table — (DOCX) [file pmed.1002916.s007.docx]

**S6 Table. Association of BMI with outcomes in the entire cohort (n=5,395)**

|  | **Number at risk** | **Number of events (%)** | **Hazard ratio (95% CI)  unadjusted** | **p-value** | **Hazard ratio (95% CI) adjusted*** | **p-value** | **p-interaction with EF** |
| --- | --- | --- | --- | --- | --- | --- | --- |
| **1 year all-cause mortality** |  |  |  |  |  |  |  |
| BMI, per 1kg/m^2^ | 5395 | 525 (9.7%) | 0.94 (0.93, 0.96) | <0.001 | 0.95 (0.92, 0.97) | <0.001 | 0.931 |
|  |  |  |  |  |  |  |  |
| BMI groups, kg/m^2^ |  |  |  |  |  |  | 0.365 |
| <18.5 | 319 | 54 (16.9%) | 2.63 (1.89, 3.67) | <0.001 | 2.41 (1.67, 3.46) | <0.001 |  |
| 18.5-23 | 1566 | 177 (11.3%) | 1.73 (1.35, 2.22) | <0.001 | 1.76 (1.34, 2.32) | <0.001 |  |
| 23-27.5 | 2055 | 196 (9.5%) | 1.43 (1.12, 1.83) | 0.004 | 1.49 (1.15, 1.94) | 0.003 |  |
| >=27.5 | 1455 | 98 (6.7%) | 1.00 (Reference) |  | 1.00 (Reference) |  |  |
| **1 year composite outcome** |  |  |  |  |  |  |  |
| BMI, per 1kg/m^2^ | 5395 | 1068 (19.8%) | 0.97 (0.96, 0.99) | <0.001 | 0.97 (0.96, 0.98) | <0.001 | 0.472 |
|  |  |  |  |  |  |  |  |
| BMI groups, kg/m^2^ |  |  |  |  |  |  | 0.369 |
| <18.5 | 319 | 86 (30.0%) | 1.60 (1.25, 2.04) | <0.001 | 1.61 (1.23, 2.10) | <0.001 |  |
| 18.5-23 | 1566 | 347 (22.2%) | 1.29 (1.10, 1.52) | 0.002 | 1.33 (1.11, 1.59) | 0.002 |  |
| 23-27.5 | 2055 | 377 (18.4%) | 1.04 (0.88, 1.21) | 0.664 | 1.08 (0.91, 1.29) | 0.351 |  |
| >=27.5 | 1455 | 258 (17.7%) | 1.00 (Reference) |  | 1.00 (Reference) |  |  |
