# Supplementary material for: Association of obesity with heart failure outcomes in 11 Asian regions: A cohort study
Source: PLoS Med. 2019 Sep 24;16(9):e1002916. doi: 10.1371/journal.pmed.1002916 (PMC6759142; doi:10.1371/journal.pmed.1002916)
Supplement: S7 Table — (DOCX) [file pmed.1002916.s008.docx]

**S7 Table. Sex-stratified analysis of waist-to-height ratio quartiles and outcomes**

| **WHtR quartiles** | **1 year mortality** | | | |  | **1 year HF hospitalization** | | | |
| --- | --- | --- | --- | --- | --- | --- | --- | --- | --- |
|  | **Number at risk** | **Number of events (%)** | **Adjusted* HR**  **(95% CI)** | **p-value** |  | **Number at risk** | **Number of events (%)** | **Adjusted* HR**  **(95% CI)** | **p-value** |
| Men |  |  |  |  |  |  |  |  |  |
| ≥0.60 | 295 | 34 (11.5%) | 1.74 (1.04,2.90) | 0.035 |  | 295 | 29 (9.8%) | 2.42 (1.36,4.29) | 0.003 |
| 0.55-<0.60 | 323 | 35 (10.8%) | 1.12 (0.71,1.77) | 0.623 |  | 323 | 22 (6.8%) | 1.28 (0.71,2.30) | 0.420 |
| 0.51-<0.55 | 379 | 42 (11.1%) | 1.0 (ref) |  |  | 379 | 23 (6.1%) | 1.0 (ref) |  |
| <0.51 | 334 | 37 (11.1%) | 0.80 (0.50, 1.26) | 0.336 |  | 334 | 21 (6.3%) | 2.42 (1.36,4.29) | 0.620 |
| Women |  |  |  |  |  |  |  |  |  |
| ≥0.60 | 158 | 21 (13.3%) | 3.31 (1.09,10.0) | 0.034 |  | 158 | 16 (10.1%) | 1.31 (0.47,3.62) | 0.603 |
| 0.55-<0.60 | 110 | 8 (7.3%) | 1.22 (0.37,4.07) | 0.747 |  | 110 | 7 (6.4%) | 0.73 (0.23,2.31) | 0.593 |
| 0.51-<0.55 | 60 | 4 (6.7%) | 1.0 (ref) |  |  | 60 | 5 (8.3%) | 1.0 (ref) |  |
| <0.51 | 87 | 9 (10.3%) | 1.17 (0.35,3.94) | 0.796 |  | 87 | 6 (6.9%) | 0.70 (0.21,2.38) | 0.569 |

*Adjusted for age, ethnicity, enrolment type, BMI and LVEF
